# Anti-Müllerian Hormone Predictive Levels to Determine The Likelihood of Ovarian Hyper-Response in Infertile Women with Polycystic Ovarian Morphology

**DOI:** 10.22074/IJFS.2020.134614

**Published:** 2021-03-11

**Authors:** Azadeh Akbari Sene, Mahnaz Ashrafi, Nasim Alaghmand-Fard, Neda Mohammadi, Mona Mortezapour Alisaraie, Ahad Alizadeh

**Affiliations:** 1 Shahid Akbar-Abadi Clinical Research Development Unit (ShACRDU), Iran University of Medical Sciences (IUMS), Tehran, Iran; 2 Department of Obstetrics and Gynaecology, Shahid Akbar-Abadi Hospital, Iran University of Medical Sciences, Tehran, Iran; 3 Department of Epidemiology and Biostatistics, School of Public Health, Tehran University of Medical Sciences, Tehran, Iran; 4 Shahid Akbar-Abadi Hospital IVF Centre, Iran University of Medical Sciences, Tehran, Iran; 5 Metabolic Diseases Research Centre, Research Institute for Prevention of Non-Communicable Diseases, Qazvin University of Medical Sciences, Qazvin, Iran

**Keywords:** Anti-Müllerian Hormone, Assisted Reproductive Technology, Body Mass Index, Ovarian Hyper-Stimula- tion Syndrome, Polycystic Ovarian Morphology

## Abstract

**Background:**

The objective of this study was to investigate serum levels of anti-Müllerian hormone (AMH) in normal-ovulatory infertile women with polycystic ovarian morphology (PCOM) and their association with ovarian hyper-response.

**Materials and Methods:**

This prospective cohort study was carried out on 100 infertile women with PCOM who were treated with an antagonist/agonist triggered stimulation protocol at Shahid Akbar-Abadi Hospital IVF Centre, Tehran, Iran. Serum AMH levels were measured before starting the assisted reproductive technology (ART) cycle and the ovarian hyper-response was evaluated by retrieved oocyte numbers, ooestradiol levels on the triggering day, and the incidence of ovarian hyper-stimulation syndrome (OHSS) clinical signs and symptoms. Logistic re- gression and the area under the curve (AUC) were used to estimate the effects of AMH and the accuracy of the test.

**Results:**

Receiver operating characteristic (ROC) curve analysis showed that AMH could significantly predict ovar- ian hyper-response in PCOM patients (AUC=0.73). The estimated threshold value was 4.95 ng/ml, with a specificity of 74.58% (95% confidence interval [CI]: 50.85, 93.22) and sensitivity of 73.17% (95% CI: 48.78, 92.68). Logistic regression results showed a significant interaction between AMH and body mass index (BMI, P=0.008), which indi- cated that BMI had a moderation effect.

**Conclusion:**

Individualized stimulation protocols for patients with isolated PCOM and AMH greater than 4.95 ng/ml may significantly reduce the chances of developing OHSS. However, the AMH cut-off values to predict ovarian hyper- response differ for different BMI categories among PCOM patients; thus, it becomes a more precise predictive marker with increasing BMI.

## Introduction

Anti-Muller hormone (AMH) is a member of the extended transforming growth factor-beta (TGF-β) family. It is secreted from the granulose cells of the small antral and pre-antral follicles in order to set the initial stages of follicular evolution (1). AMH is a hormone biomarker that is suitable for evaluating the follicular numbers of the ovary; its serum levels indirectly show ovarian reserve (2). AMH levels are independent of the hypothalamus-pituitary axis (3). Therefore, there is little variation during a menstrual cycle and at intervals between cycles (4). AMH serum levels are closely related to the number of primary antral follicles in healthy women and those with polycystic ovary syndrome (PCOS) (5). A decreased AMH level indicates low ovarian reserve; consequently, elevated serum levels indicate increased ovarian reserve and, although it can be a valuable tool for PCOS detection (6), there are many limitations for its use as a PCOS diagnostic tool. The Rotterdam criteria define PCOS as the most common endocrinopathy in women of reproductive age, with the presence of two of the following conditions: Oligoovulation or non-ovulation, clinical or laboratory hyperandrogenism, and polycystic ovarian morphology (PCOM) visualized on ultrasound. Failure of follicular maturation in patients with PCOS leads to non-ovulation, and accumulation of pre-antral and antral follicles; this is clearly associated with increased AMH secretion (7). In assisted reproductive technology (ART) cycles, infertile women with PCOS have a higher incidence of ovarian hyper-stimulation syndrome (OHSS), and it is a potential iatrogenic and potentially life-threatening problem (8, 9).

OHSS is the consequence of vasoactive mediators being released from hyper stimulated ovaries. Thus, increased capillary permeability causes extravasation of fluid from the intravascular compartment into the third space. “The haemoconcentration which ensues results in complications such as hypercoagulability and reduced end organ perfusion” (10-12). Young age, low body weight, PCOS, and a previous history of OHSS are known risk factors for OHSS (13-15). Hormonal biomarkers are used to predict the ovarian response to stimulation and AMH is a measurement that shows tremendous promise (10). On the other hand, a group of healthy women with regular menstrual cycles and normal ovulation, and who lack clinical or laboratory evidence of hyperandrogenism, are also candidates for ART. In these women, PCOM is only visible by ultrasonography. PCOM is the presence of at least one ovary with 12 or more follicles, 2 to 10 mm in a single plane, or a volume of ovaries greater than 10 ml in the absence of a dominant follicle greater than 10 mm, lupus corpus luteum or cyst. This condition is seen in the absence of PCOS in 25% of normal women (16).

The primary outcome of this study was to evaluate the predictive level of AMH to determine the likelihood of an ovarian hyper-response among normal-ovulatory infertile women with PCOM. According to recent data about the effects of body mass index (BMI) on AMH levels (17-19), our secondary objective was to investigate the AMH cut-off levels in different BMI categories among women with PCOM.

## Materials and Methods

### Study population

This prospective cohort study was carried out on 100 infertile women with PCOM who referred to the IVF Centre of Shahid Akbar-Abadi Hospital in Tehran, Iran. The women were between 20 and 40 years of age, and were candidates for ART with tubal or male factor. All participants had regular menstruation, no history or symptoms of clinical or laboratory evidence of hyperandrogenism (hirsutism, acne, balding) and hyperandrogenemia (normal levels of serum testosterone, Dehydroepiandrosterone sulfate (DHEAS) and 17 OH progesterone in the early follicular stage). PCOM was defined according to the International evidence-based guideline for the assessment and management of PCOS (20) as women with an ovarian volume ≥10 ml for either ovary at an endovaginal ultrasound assessment.

Women with the following characteristics were excluded: less than 20 and over 40 years of age, thyroid disorders or hyperprolactinaemia, premature ovarian failure, abnormal karyotype, and clinical or laboratory hyperandrogenism. The Ethics Committee of Iran University of Medical Sciences approved this study. The study was registered with the code: CIR.IUMS.RE 1394.92190025711 and all participants signed a written informed consent. At the beginning of each cycle, demographic characteristics that included age and BMI were recorded. Serum AMH levels were measured with an Anti-Müllerian Hormone Gen II Enzyme-linked ImmunosorbentAssay (ELISA) kit (Beckman Coulter Immunotech, USA). The lowest detection rate limit that had a 95% probability was 0.08 ng/ml.

In the antagonist cycle, patients were monitored by sonography on the second day of the menstrual cycle. The patients received recombinant follicle stimulating hormone (FSH) at a dose of 150 units per day (Gonal-F®; Merck, Geneva, Switzerland) and follicular growth was monitored by vaginal ultrasonography. When the follicular diameters reached 13-14 mm, the patient began daily administration of 0.25 mg gonadotropin-releasing hormone (GnRH) antagonist (Cetrotide 0.25 mg, Merck Serono, Germany). Triggering was performed with a 0.2 mg GnRH-Agonist injection (Decapeptyl 0.1 mg, Ferring Pharmaceuticals) when there were at least three, 17 mm follicles. Serum ooestradiol levels were measured on the triggering day and ovarian puncture was performed after 36 hours. The numbers of oocytes and the presence and severity of OHSS clinical symptoms were documented. All embryos were frozen until transfer in subsequent embryo transfer (FET) cycles. A patient was considered to be a hyper-responder when a triggering day ooestradiol level was more than 3500 pg/dl, and/or the retrieved oocytes was more than 15 (21), and/orclinical manifestations of OHSS (based on Navot’s criteria) were present (22).

### Statistical analysis

Data were analysed using R software version 3.4.1, “pROC”, “plotROC”, “verification”, “Resource Selection”, “multcomp”, and “ggplot2” packages (23-25). Primary descriptive results were reported using median and inter quartile range (IQR) for quantitative non-parametric variables, mean ± standard deviation (SD) for normal variables, and number (percent) for qualitative variables. Normality of the quantitative variables were assessed by the Lilliefors test. The Mann-Whitney U or independent sample t tests were used to compare the distribution of quantitative variables or mean as appropriate. The association of qualitative variables was evaluated using the chi-square test. P values were estimated based on 10000 sampled tables by the Monte Carlo method.

Binary logistic regression was used to estimate the effects of AMH and other factors on hyper-response. Outputs of this method were reported using odds ratio (OR) and 95% confidence interval (95% CI). Receiver operating characteristic (ROC) analysis was used to evaluate the prediction performance of AMH. The accuracy of the test was estimated by the area under the curve (AUC) and the CI of that was calculated using the DeLong method. The Youden index (J) was usedto obtain the best cut-off points and the clinical diagnostic ability of AMH (26). This index is defined as J=max [sensitivity (j)+specificity (j)-1], where “j” is the cut-off point. It is a popular measurement for ROC curve analysis and an optimal trade-off between sensitivity and specificity (27). The sensitivity and specificity CI were computed with 2000 stratified bootstrap replicates. A total sample size of 100 achieved an 86% power to detect a change in sensitivity from 50 to 74.58% using a two-sided binomial test and 95% power to detect a change in specificity from 50 to 73.17%. The level of significant was set at P<0.05.

## Results

We analysed data from 100 infertile patients with PCOM to determine the performance of AMH as a biomarker for hyper-response during* in vitro* fertilization (IVF) cycles. Table 1 presents the demographic and biochemical baseline characteristics, and the controlled ovarian stimulation (COS) outcome of PCOM patients with and without ovarian hyper-response. Hyper-response after COS was defined as retrieved oocyte numbers >15 and/or ooestradiol level on the triggering day >3500 pg/ml. In total, 41% (n=41) of the PCOM patients met the criteria for ovarian hyper-response. There were no cases of moderate, severe or critical OHSS according to the GnRH antagonist/agonist triggered/freeze all protocol and Navot’s criteria (22). In the hyper-responder group, 20 (48.8%) patients had mild clinical manifestations of hyper-response, which included nausea and/or bloating, and were symptomatically managed as outpatients. 

The median number of oocytes in the suboptimal/normal responder group was 8, and there were 20 in the hyper-responder group, which was statistically significant (P<0.001). The serum ooestradiol level in the hyperresponder group increased dramatically on the triggering day (P<0.001). In addition, patients in the hyper-responder group had a significantly lower average BMI compared to the suboptimal/normal responder group (P=0.027). There was a difference in the median AMH levels between the two groups, which suggested that AMH positively affected the level of ovarian response (P=0.002).

The main aim of the present study was to evaluate the performance and accuracy of AMH as a clinical predictor for the likelihood of ovarian hyper-response during ovarian stimulation in ART cycles in patients with PCOM. According to a crude analysis by logistic regression, the odds of hyper-responsiveness increased 1.28-fold with each ng/ml increase in the level of AMH (OR=1.28, 95% CI: [1.11, 1.5], P=0.001, Table 2). Interestingly, the Hosmer-Leme show test, as a statistical method to evaluate the goodness of fit of a model, was not significant, which indicated that AMH was an appropriate biomarker to predict ovarian response in patients with PCOM during IVF cycles (chi-square: 9.76, degree of freedom: 8, P=0.28). 

**Table 1:** Demographical and biochemical baseline characteristics and COS outcomes of PCOM patients with and without ovarian hyper-response


Factors		PCOM women without ovarian hyper-response after COS n=59	PCOM women with ovarian hyper-response after COS n=41	P value

Characteristic data				
Age (Y)		31.02 ± 4.29	30.59 ± 5.89	0.690
FSH (IU/ml)		5.54 ± 2.61	5.93 ± 3.10	0.474
Total gonadotropin dose (IU)		2800 ± 101.54	1825 ± 48.35	<0.001
Duration of stimulation (Days)		12.5 ± 1.3	12.15 ± 1.1	0.134
AMH (ng/ml), median (IQR)		3.8 (3.15, 5.45)	6.8 (4.8, 8.8)	0.002
BMI (kg/m^2^)		27.82 ± 3.80	26.06 ± 3.92	0.027
BMI categories (kg/m^2^)				0.001
	<25	9 (15.3)	20 (48.8)	
	25-30	35 (59.3)	11 (26.8)	
	≥30	15 (25.4)	10 (24.4)	
COS outcomes				
Number of follicles on triggering day, median (IQR)		8 (5, 10)	20 (16, 27)	<0.001
Number of follicles on triggering day based on ovarian response				<0.001
	Poorresponse (0-3)	5 (8.47)	0 (0)	
	Suboptimalresponse (4-9)	36 (61.02)	0 (0)	
	Normalresponse (10-15)	18 (30.51)	0 (0)	
	Hyper-response (>15)	0 (0)	41 (100)	
Oestradiol level on triggering day (pg/ml), median (IQR)		1590 (904.5, 2252)	6768 (2710, 9000)	<0.001


Data are presented as mean ± SD or n (%). PCOM; Polycystic ovarian morphology, COS; Controlled ovarian stimulation, AMH; Anti-Müllerian hormone, FSH; Follicle stimulating hormone, BMI; Body mass index, and Ovarian hyper-response; Retrieved oocytes>15 and/or ooestradiol level on triggering day>3500 pg/ml.

**Table 2:** Evaluation and estimation of the prediction performance and effects of AMH and BMI by using univariate and multivariate logistic regressions and analysis of the AUC


Factors	Univariate analysis			Multivariate Analysis		
	OR	P value	AUC of model (95% CI)^*^^*^	AOR (95% CI)	P value	AUC of model (95% CI)^*^^*^

AMH (ng/ml)	1.28 (1.11, 1.5)	0.001	0.73 (0.63, 0.84)	1.17 (0.91, 1.59)	0.264	0.82 (0.74, 0.91)
BMI (<25 kg/m^2^)^*^	1	-	0.71 (0.61, 0.81)	1	-	
BMI (25-30 kg/m^2^)	0.14 (0.05, 0.39)	<0.001		0.14 (0.01, 1.6)	0.113	
BMI (≥30 kg/m^2^)	0.3 (0.09, 0.9)	0.035		0.01 (0, 0.37)	0.026	
Increase of 1 ng/ml AMH in BMI (25-30 kg/m^2^) to BMI (<25 kg/m^2^)	-	-	-	1.02 (0.71, 1.43)	0.911	
Increase of 1 ng/ml AMH in BMI (≥30 kg/m^2^) to BMI (<25 kg/m^2^)	-	-	-	2.38 (1.19, 6.62)	0.035	


OR; Odds ratio, AOR; Adjusted odds ratio, CI; Confidence interval, AUC; Area under the curve (calculated by predicted values of logistic regression), BMI; Body mass index, AMH; AntiMüllerian hormone, * ; Reference level, and **; Confidence interval was calculated using the DeLong method.

**Table 3:** Estimation of the best cut-off points for AMH in the total samples (overall) and according to BMI


Class	Threshold (95% CI)	Specificity (95% CI)	Sensitivity (95% CI)

Overall	4.95 (3.85, 6.6)	74.58 (50.85, 93.22)	73.17 (48.78, 92.68)
BMI (kg/m^2^)			
<25	9.8 (4.65, 10.3)	100 (55.56, 100)	50 (20, 95)
25-30	5.45 (5, 8.05)	77.14 (60, 94.29)	81.82 (54.55, 100)
≥30	3.85 (2.65, 5.9)	86.67 (53.33, 100)	90 (50, 100)


AMH; Anti-Müllerian hormone, BMI; Body mass index, and CI; Confidence interval.

**Fig.1:**
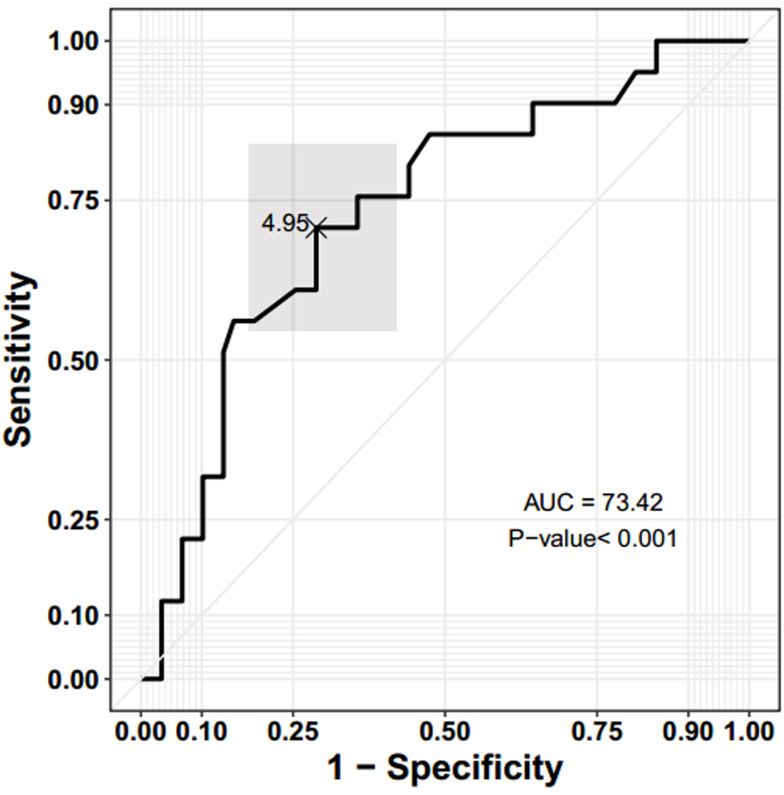
Receiver operating characteristic (ROC) curve of anti-Müllerian hormone (AMH). The point, “×”, refers to the best cut-off point, which is estimated by Youden’s index (J). The gray rectangle refers to a 95% bivariate confidence interval (CI) of sensitivity and 1-specificity. AUC; Area under the ROC Curve.

ROC curve analysis showed that AMH had a significant performance to assign the PCOM patients to their true status of hyper- and normal responder groups. The AUC was equal to 0.73, which indicated a reasonable accuracy of this test, and it was statistically different from a test that randomly assigned patients to the groups (AUC: 0.73, 95% CI: [0.63, 0.83], P<0.001). In other words, 73% of patients were correctly assigned to the suboptimal/normal responder or hyper-responder groups by AMH. Figure 1 shows the ROC curve of the AMH marker. The multiplication sign in this figure refers to the best cut-off point, which was estimated by Youden’s index (J) (threshold value: 4.95, 95% CI: [3.85, 6.60]). According to the estimated threshold value by Youden’s index (J), AMH had a specificity of 74.58% (95% CI: [50.85%, 93.22%]) and a sensitivity of 73.17% (95% CI: [48.78%, 92.68%], Table 3, first row).

**Fig.2:**
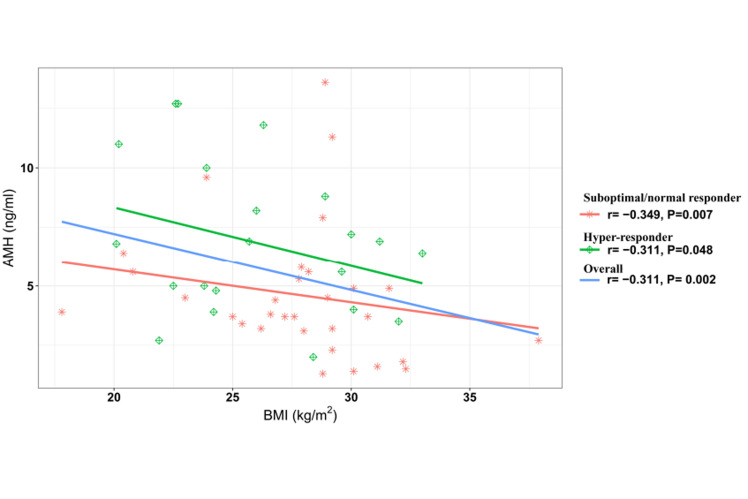
Correlation and linear trend lines of BMI and AMH base on ovarian response groups and total patients with PCOM (overall). BMI; Body mass index, AMH; Anti-Müllerian hormone, and PCOM; Polycystic ovarian morphology.

Correlation analysis of BMI and AMH showed an inverse correlation between these variables in the hyper-responder (r=-0.311, P=0.048) and the suboptimal/normal responder (r=-0.349, P=0.007) groups. In general, there was a significant negative correlation between AMH and BMI in the PCOM patients (r=-0.311, P=0.002, Fig.2).This negative correlation showed that different values of BMI could moderate the behaviour of AMH as a biomarker for prediction of ovarian hyper-response.

BMI is classified into three groups based on the WHO classification -<25 kg/m^2^ , 25-30 kg/m^2^ , and>30 kg/m^2^ . Crude analysis by logistic regression showed a positive association between an increasing crude AMH and a higher risk of hyper-response (Table 2). Conversely, the association of BMI and a higher risk of hyper-response were significantly negative. In other words, the odds of a hyper-response for a patient with a BMI from 25-30 kg/ m^2^ was 0.14-fold less than a patient with a BMI of <25 kg/ m^2^ (OR: 0.14, 95% CI: [0.05, 0.39], P<0.001). Additionally, the odds of a hyper-response in a patient with a BMI >30 kg/m^2^ was 0.3-fold less than a patient with a BMI of <25 kg/m^2^ (OR: 0.3, 95% CI: [0.09, 0.9], P=0.035). 

Table 2 shows the results of multivariate logistic regression, which estimated the effects of AMH, BMI, and their interactions. The effect of AMH on ovarian hyper-response at different BMI levels did not have the same slope because of an existing significant interaction between AMH and BMI (deviance of likelihood ratio test: 7.51, degree of freedom: 2, P=0.023). Therefore, it was necessary to separately consider the relationship of AMH and hyper-response in each BMI subgroup. There was an approximately 2.38-fold increase in the odds of developing a hyper-response with each one ng/ml increase of AMH in patients with BMI ≥30 kg/m^2^ compared to a BMI <25 kg/m^2^ (OR: 2.38, 95% CI: [1.19, 6.62], P=0.035, Table 2).

**Fig.3:**
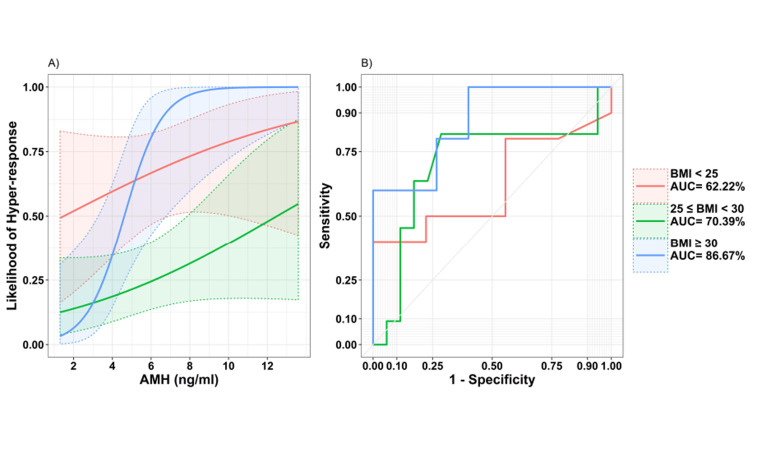
The likelihood of hyper-response and ROC curve analysis. A. The likelihood of hyper-response against AMH based on BMI groups and B. ROC curve of AMH in the three BMI groups. AMH; Anti-Müllerian hormone, BMI; Body mass index, ROC; Receiver operating characteristic, and AUC; Area under the ROC curve.

Figure 3A shows the behaviour of the interaction effect. In this figure, the probability of developing a hyper-response is shown against the increase in AMH based on the BMI groups. Patients with a BMI<25 kg/m^2^ had the highest probability of developing a hyper-response when the AMH values were less than approximately 5 ng/ml; however, with values greater than 5 ng/ml, the probability of a hyper-response was highest in the BMI>30 kg/m^2^ group. Based on Figure 3A, a woman with a BMI >30 kg/ m^2^ and an AMH level over approximately 10 ng/ml was completely at risk for ovarian hyper-response (likelihood ≈ 1). Overall, this chart shows that an increase in AMH increases the probability of developing a hyper-response in all three BMI groups; however, this increase is much steeper in PCOM patients with a BMI≥30 kg/m^2^ .

According to the BMI classification, ROC curves for AMH showed that the accuracy of AMH for predicting hyper-responsiveness in all three classes of BMI constantly increased (Fig.3B). Advanced analysis revealed that there were different cut-off points for AMH according to BMI classification (Table 3). These results were estimated using Youden’s index (J) and they were consistent with the previous logistic regression results.

## Discussion

Serum AMH level is an indirect reflection of the ovarian follicular reserve and, therefore, many researchers consider it to be a sensitive biomarker of ovarian aging and ovarian reserve (2, 8). AMH serum levels are closely correlated with the number of early antral follicles in both healthy women and women with PCOS (5, 28), and it is mostly produced by granulosa cells of follicles from 2 to 9 mm in diameter. Impaired folliculogenesis in PCOS patients may cause excess accumulation of pre-antral and small antral follicles, which may ultimately lead to an increase in AMH levels. The results of numerous studies show elevated AMH levels in PCOS patients. Although there is no worldwide standard for serum AMH assays and, thus, no defined thresholds, it has been suggested that a hyper-response or OHSS might be anticipated at approximately 3.5  ng/ml or higher during ART cycles (6, 8, 29).

In our study, we investigated the role of AMH as a predictor of ovarian hyper-response in a specific group of infertile women with PCOM. These women had regular menstrual cycles and normal ovulation, and no hyperandrogenism. However, they had PCOM on ultrasound examination. We observed that AMH levels in our PCOM hyper-responders (based on a triggering day oestradiol of >3500 pg/dl, and/or >15 retrieved oocytes, and/or clinical manifestations of OHSS) were significantly higher than in the PCOM suboptimal/normal responder group. In addition, with each one ng/ml increase in the AMH level, the risk of hyper-responsiveness increased by 1.28 fold.

Different studies have calculated various AMH cut-off values for hyper-response in non-PCOS infertile women and in patients with PCOS. Vembu and Reddy (9), in their study of 246 women (31% PCOS and 78% non-PCOS), suggested a cut-off value of 6.85 ng/ml with a sensitivity of 66.7% and a specificity of 68.7% in PCOS patients and 4.85 ng/ml with a sensitivity of 85.7% and a specificity of 89.7% in non-PCOS patients to predict a hyper-response. On the other hand, Zhang et al. (30) proposeda lower cut-off value to predict ovarian hyper-response among their 120 PCOS patients - 2.84 ng/ml with a sensitivity of 72.7% and specificity of 65.9%. Mahajan and Kaur (31) reported that the mean AMH level of Indian women with PCOS was higher (7.56 ± 4.36 ng/mL) than women with isolated PCOM and controls. They reported that serum AMH concentrations over 5.03 ng/mL could predict the PCOS (AUC: 0.826; sensitivity: 70.68%, and specificity: 79.91%). These differences in AMH threshold could be related to the lack of a well-defined population, stability and heterogeneity of circulating AMH, a wide range of reference values, inter-laboratory variability, and different immunoassays used worldwide (6).

We have calculated an AMH threshold specifically for a normal-ovulatory subgroup of infertile women with PCOM. According to our literature reviews, this has not been investigated. The risk of hyper-response increased in our studied PCOM patients at an AMH cut-off of 4.95 ng/ ml, which had a specificity of 74.58% and a sensitivity of 73.17%. Because of the relatively limited numbers in our studied population, further studies with a larger number of PCOM patients are required to develop a more precise cut-off value. However, based on our findings, we suggest that it is possible to tailor a safe stimulation protocol for normal-ovulatory infertile patients who have a polycystic ovarian appearance and an AMH level over 4.95 ng/ml.

Of note, we had a group of poor/suboptimal-responders among our PCOM patients. Despite the increased antral folliculate count, the low follicle output rate (FORT) in this group of patients might be related to a hypo-sensitivity/ hypo-response to FSH due to genetic characteristics like FSH receptor polymorphism or luteinizing hormone-beta (LH-beta) variants (32)

The average BMI in our PCOM hyper-responder group was significantly lower than among the suboptimal/normal responder group. According to univariate analysis, the association between BMI and a high risk of hyper-response was significantly negative. On the other hand, the correlation analysis of BMI and AMH showed an inverse correlation between these two variables among both hyper-responder and suboptimal/normal responder PCOM patients. The correlation between AMH and BMI has been investigated by other studies and the results are controversial.

In a retrospective study of 951 non-PCOS women, Simões-Pereira et al. (33) did not observe any significant effect of BMI on AMH levels. In another retrospective cohort study, Kriseman et al. (34) did not find any association between BMI and AMH levels in a general population of infertile women or in patients without PCOS. However, the BMI was significantly and inversely correlated with AMH among their 104 PCOS patients. In addition, Lefebvre et al. (35) studied 691 women and found no effect of metabolic status on serum AMH levels in the non-PCOS group; however, there was a significant, albeit weak, negative independent correlation between AMH and BMI for women with PCOS. Moy et al. (36) reported a negative correlation between elevated BMI and AMH in Caucasian women, but not in African-American, Hispanic, or Asian women. They suggested further studies should be conducted to evaluate the effect of race on the interaction between obesity and ovarian reserve.

A recent meta-analysis showed that the AMH level was significantly lower in obese compared to non-obese reproductive-aged women, and BMI had a negative correlation with AMH in PCOS and non-PCOS subjects. The authors concluded that PCOS and fertility status do not appear to affect this association (18). Interestingly, weight loss in adolescent girls with PCOS has been found to be associated with a significant drop in AMH concentrations, and the hormone level becomes normalized (17). NilssonCondori et al. (19) also observed that AMH levels increased in 48 young obese women who were placed on a very lowcalorie diet prior to bariatric surgery. Their AMH levels decreased at 6 and 12 months after Roux-en-Y gastric bypass, and this decrease was beyond the expected normal age-related decline. However, they did not evaluate their subjects for ovarian morphology and PCOS. A negative impact of BMI on AMH levels has been reported among women with diminished ovarian reserve (37).

We hypothesized that the negative correlation between AMH and BMI could change the behaviour of AMH as a biomarker in predicting an ovarian hyper-response in the presence of different values of BMI. BMI is also a possible predictive factor for ART outcomes, so it could be confounded with the relationship between AMH and an ovarian hyper-response. Our multivariate logistic regression analysis revealed that a significant interaction existed between AMH and BMI on ovarian hyperresponse. In general, we observed an increase in the AMH level, which increased the probability of developing a hyper-response in all BMI groups. This increase was more prominent in PCOM patients who had a BMI over 30 kg/m^2^ . Consequently, the accuracy of AMH for predicting ovarian hyper-response in the three classes of BMI constantly increased and there were different cut-off values for AMH due to the BMI classification in PCOM patients.

This finding suggests that the behaviour of serum AMH levels, as a predictive biomarker for ovarian response, might be more complicated in PCOM patients who have a higher BMI and it may not accurately present the true ovarian capacity to develop an exaggerated response in obese patients. Although there is no clear explanation for this issue, one possible explanation could be the positive correlation between AMH and LH levels (38). LH levels are suppressed in obese women due to increased peripheral aromatization and oestrogen production in fat tissue, which may result in lower serum AMH levels in these patients (39). Recently, it has been demonstrated that serum AMH levels are positively correlated with antral follicular count. They are also positively correlated with serum LH and free testosterone levels, and negatively correlated with total body fat and percent body fat in PCOS patients (40). In addition, it has been suggested that obesity may affect the catabolism of AMH. These correlations have not been investigated in the present study but should be investigated in future randomised clinical trials (RCTs). It would be interesting to study the AMH predictive values for ovarian responses in relation with other predictive factors such as BMI in other groups of infertile women, especially among the different subtypes of PCOS patients. These findings would be beneficial to develop an individualized COS programme for each infertile woman.

## Conclusion

The findings of the present study suggest that infertile normal-ovulatory women with PCOM are at risk of an ovarian hyper-response at AMH levels greater than 4.95 ng/ml. For this reason, individualized stimulation protocols for this group of patients with PCOM and AMH greater than 4.95 ng/ml may significantly reduce the chances of developing established moderate or severe forms of OHSS. The use of lower starting doses of gonadotropins, antagonist/agonist triggered stimulation protocols, and freezing all embryos are proposed to be effective strategies to achieve this goal. However, based on our findings, women with PCOM and AMH levels lower than 4.95 ng/ml are not considered high risk for hyper-response. The use of other stimulation protocols and fresh embryo transfer would be considered appropriate for them.

The AMH cut-off values to predict ovarian hyper-response are different for different BMI categories among PCOM patients; thus AMH becomes a more precise predictive marker as the BMI increases. It would be valuable to consider the AMH cut-off values for different BMI categories in order to develop an individually tailored, effective, and safe stimulation programme for infertile women with PCOM.
